# The association of total oxidant status, total antioxidant status, and ischemia-modified albumin levels with cervical human papillomavirus infection and cervical intraepithelial neoplasia

**DOI:** 10.1590/1806-9282.20252260

**Published:** 2026-07-31

**Authors:** Belgin Savran Ucok, Tugba Kinay, Ilknur Alkan Kusabbi, Murat Levent Dereli, Sadun Sucu, Turkan Dikici Aktas, Emel Ozalp, Ozgur Volkan Akbulut, Vakkas Korkmaz, Yaprak Engin Ustun

**Affiliations:** 1 University of Health Sciences, Ankara Etlik City Hospital, Department of Obstetrics and Gynecology – Ankara, Turkey.; 2 University of Health Sciences, Ankara Etlik City Hospital, Department of Medical Biochemistry – Ankara, Turkey.; 3 Denizli State Hospital, Department of Obstetrics and Gynecology – Denizli, Turkey.; 4 Eskisehir City Hospital, Department of Obstetrics and Gynecology – Eskişehir, Turkey.; 5 Lokman Hekim University, Ankara Hospital, Department of Obstetrics and Gynecology – Ankara, Turkey.; 6 Izmir City Hospital, Department of Obstetrics and Gynecology – İzmir, Turkey.

**Keywords:** Cervical intraepithelial neoplasia, Human papillomavirus, Oxidative stress, Antioxidant, Ischemia-modified albumin

## Abstract

**OBJECTIVE:**

The aim of this study was to evaluate the association between oxidative stress and cervical human papillomavirus infection and cervical intraepithelial neoplasia by measuring total oxidant status, total antioxidant status, and ischemia-modified albumin levels.

**METHODS:**

In total, 50 human papillomavirus+ women, 50 cervical intraepithelial neoplasia2/3+ women, and 50 healthy women as a control group were enrolled in the prospective case–control study. The total oxidant status, total antioxidant status, and ischemia-modified albumin levels were measured with colorimetric systems. The clinical characteristics, total oxidant status, total antioxidant status, and ischemia-modified albumin levels of three groups were compared.

**RESULTS:**

The mean total antioxidant status level was higher in the control group (1.044±0.0959 mmol/L) than in the human papillomavirus+ group (0.931±0.1034 mmol/L) and the cervical intraepithelial neoplasia2/3+ group (0.927±0.0928 mmol/L) (p<0.001). The median total oxidant status level was lower in the control group than the cervical intraepithelial neoplasia2/3+ group (4.570 [4.390–4.783] μmol/L vs. 4.780 [4.443–5.035] μmol/L, p<0.05). The ischemia-modified albumin levels were similar in three groups (p=0.220).

**CONCLUSION:**

The total antioxidant status level was lower, and the total oxidant status level was higher in women with cervical intraepithelial neoplasia2/3+ compared to the control group. These findings suggest that oxidative stress is effective in the progression of human papillomavirus infection to the preinvasive lesions of the cervix. The ischemia-modified albumin was not found to be a useful biomarker to indicate oxidative stress in women with human papillomavirus infection or cervical intraepithelial neoplasia2/3 lesions.

## INTRODUCTION

Cervical cancer is the fourth most common cancer worldwide after breast, colorectal, and lung cancer among women^1^. Cervical cancer is a rare long-term consequence of persistent infection of the lower genital tract with the high-risk human papillomavirus (HPV) types. About 10% of HPV infections become persistent, and these women may develop cervical intraepithelial neoplasia (CIN) and/or cancer^2^. The factors that lead to persistent HPV infection, and the progression to CIN and cervical cancer have not yet been fully understood. It has been suggested that various viral, host, and environmental cofactors may contribute to progression to neoplasia. Oxidative stress is thought to be one of these cofactors. The chronic inflammatory environment caused by persistent HPV infection forces the formation of reactive oxygen species, causing genomic instability, DNA damage, and malignant transformation in cervical cells^3^.

Previous studies reported the association between cervical cancer and oxidative stress^4,5^. However, there are limited studies reporting the association between HPV infection, progression to preinvasive lesions, and oxidative biomarkers^6,7,8^. In these studies, various oxidant and antioxidant biomarkers were measured separately with time-consuming and complicated techniques. Total oxidant status (TOS) and total antioxidant status (TAS) are the biomarkers that can be measured with simple and inexpensive methods and show the body’s overall oxidative status. The TOS measurement principle was based on the oxidation of the ferrous ion–chelator complex to ferric ions^9^, and the TAS measurement principle was based on the reduction of 2,2’-azinobis-3-etilbenzotiazolin-6-sülfonat (ABTS) radicals^10^. Ischemia-modified albumin (IMA), which is the modified form of the N-terminal of albumin and has a reduced metal-binding capacity under conditions of free radical damage, energy-related membrane damage, acidosis, and hypoxia, is also a biomarker indicating oxidative stress^11,12^. Limited studies reported the IMA levels in women with cervical preinvasive lesions and cancer^5,7^. The aim of the study was to evaluate the association between the HPV infection, progression to CIN 2/3, and oxidative stress by measuring TOS, TAS, and IMA levels.

## METHODS

The prospective case–control study was conducted between May 2025 and October 2025 at the Gynecology and Gynecological Oncology departments of a tertiary care center. The study protocol was approved by the Ethical Committee of the University of Health Sciences Ankara Etlik City Hospital (Date: 30/04/2025, Number: AEŞH-BADEK1-2025-021). Signed informed consent was obtained from all participants.

Women aged >18 years old and older who underwent an HPV test and cytology were included in the study. Women with a history of smoking, alcohol use, antioxidant vitamin use (vitamin C, vitamin E), autoimmune and/or chronic inflammatory disease, anti-inflammatory drug use, liver disease, kidney disease, malignancy, diabetes mellitus, thyroid disease, lower or upper genitourinary infections, and previous treatment for HPV infection or CIN were excluded from the study. Lower and upper genitourinary infection was diagnosed based on symptoms (pelvic pain, flank pain, vaginal discharge, dysuria, hematuria, etc.), physical and gynecological examination results (costovertebral tenderness, cervical tenderness, adnexal tenderness, leukorrhea), urinalysis, white blood cell count, C-reactive protein level, urine culture, vaginal culture, and ultrasonography results.

Demographic/clinical characteristics, HPV test, and cytology test results of the women were obtained from medical records. HPV tests were performed with Cobas HPV Kit (Roche Molecular Systems, NJ, USA) using amplification of target DNA by polymerase chain reaction (PCR) and nucleic acid hybridization to detect 14 high-risk HPV (hrHPV) types. Colposcopy was performed when abnormal HPV test and cytology results were detected. Cervical biopsy and histopathological examination were performed when abnormal findings were observed during the colposcopy procedure. Cold knife conization and loop electrosurgical excisional procedure (LEEP) were performed if indicated. The CIN2/3 was diagnosed based on cervical biopsy, cold knife conization, and LEEP results. According to the HPV test, cytology, and histopathological results, the study population was divided into three groups: the HPV+ group, women with any type of cervical HPV infection but without CIN 2/3; the CIN2/3+ group, women with HPV infection and CIN 2/3; and the control group, women with negative HPV test and cytology results. The clinical characteristics, TAS, TOS, and IMA levels of the three groups were compared.

The blood samples were drawn from the women included in the study, centrifuged at 3,000 rpm for 10 min, and then 1.5 mL serum samples were separated into Eppendorf tubes. The serum samples were kept at -80 °C until the day of the laboratory assessment. TOS, TAS, and IMA levels were determined. The measurements were carried out by applying the TAS, TOS, and IMA kits of Rel Assay Diagnostics (Turkey) to the Roche Cobas instrument. The results of TAS and TOS were stated as mmol Trolox Equiv./L and μmol H_2_O_2_ Equiv./L, respectively.

IMA levels were measured by the albumin cobalt-binding colorimetric method. In the IMA test, normal albumin existed as an active form in serum or plasma, N-terminal of albumin combined with Co+, while adding cobalt chloride solution, then the concentration of free Co+ in the solution decreased. The higher concentration of free Co+ formed more colored products when adding dithiothreitol (DTT). In a colorimetric test at a specific wavelength (sub/main; 800/470 nm), the absorbance was proportional to the concentration of free Co+. The IMA concentration in the sample was calculated by comparing the calibrator result with the same processing. The results of IMA were stated as ABSU (absorbance units).

In the power analysis performed with the G*POWER 3.1.9.7 program, when a 5% margin of error, 90% power, and 0.689 effect size were set, the minimum number of samples was calculated as 138 in total, 46 in each group^13^.

All statistical analyses were performed using the RStudio integrated development environment for statistical computing (Affero General Public License v3; published 2011). RStudio for Linux, version v2021.09.4+403.pro3 Ghost Orchid (September 19, 2022; developed by Posit, PBC.) was used to analyze the data. The variables were investigated using visual (histogram, probability plots) and analytic methods (Kolmogorov-Smirnov/Shapiro-Wilk’s test) to determine whether or not they are normally distributed. The Levene test was used to assess the homogeneity of the variance. Descriptive analyses were presented using means and standard deviation or medians and quartiles according to the normal distribution of continuous variables. One-way ANOVA, independent sample t-test, Kruskal-Wallis test, and the Mann-Whitney U test were used to compare continuous parameters. Descriptive analyses were presented using frequency and percentage for the categorical variables. Relationships between categorical variables were analyzed with the chi-square test or Fisher’s exact test. A p-value of less than 0.05 was considered to show a statistically significant result.

## RESULTS

A total of 150 women who met the inclusion criteria were included in the study. According to the HPV test, cytology, and histopathology results, the study population was divided into three groups. Demographic and clinical characteristics of groups are shown in Table 1. The mean age of the control group (37±6.2 years) was lower than the HPV+ group (41±9.1 years) and the CIN2/3+ group (43±8.7 years) (p=0.003). The median vaginal delivery number of the HPV+, CIN2/3+, and control groups were 1 (0–1), 1 (0–1), and 0 (0–1), respectively (p=0.031). Most of the study population was at reproductive age.

**Table 1:** Demographic and clinical characteristics of groups.

	HPV+ (n=50)	CIN2/3+ (n=50)	Control (n=50)	p-value
Age, years	43±8.7	41±9.1	37±6.2	0.003^a^
BMI, kg/m^2^	27 (25–29)	26 (22–29)	26 (23–28)	0.153
Parity				
Nulliparous	2 (4)	10 (20)	7 (14)	0.095
Primiparous	5 (10)	6 (12)	9 (18)	
Multiparous	43 (86)	34 (68)	34 (68)	
Vaginal delivery	1 (0–1)	1 (0–1)	0 (0–1)	0.031
Cesarean delivery	0 (0–1)	0 (0–1)	1 (0–1)	0.118
Menopausal status				
Reproductive age	23 (46)	34 (68)	38 (76)	0.007
Perimenopausal	13 (26)	8 (16)	10 (20)	
Postmenopausal	14 (28)	8 (16)	2 (4)	
Contraceptive method used				
None	24 (48)	20 (40)	19 (38)	0.118
IUD	8 (16)	12 (24)	4 (8)	
Condom	6 (12)	6 (12)	12 (24)	
Fallopian tube ligation	3 (6)	7 (14)	3 (6)	
Coitus interruptus	5 (10)	2 (4)	3 (6)	
Hormonal contraception	4 (8)	3 (6)	9 (18)	

BMI: body mass index; IUD: intrauterine device; HPV: human papillomavirus; CIN: cervical intraepithelial neoplasia. Values are given as mean±SD, median (range), or number (percentage).

^a^Comparison of groups 1 and 2 (p=0.113); comparison of groups 1 and 3 (p=0.001); comparison of groups 2 and 3 (p=0.056).

The HPV test, cytology, and histopathology results of the HPV+ group and the CIN2/3+ group are shown in Table 2. The positive HPV16/18 was observed in 36 of 50 (72%) women in the HPV+ group and 41 of 50 (82%) women in the CIN2/3+ group. Multiple HPV types were observed in 28% of the HPV+ group and 26% of the CIN2/3+ group. While ASCUS (atypical squamous cells of undetermined significance) (32%) was the most common cytology result in the HPV+ group, LSIL (low-grade squamous intraepithelial lesion) (22%) and HSIL (high-grade squamous intraepithelial lesion) (20%) were more commonly observed in the CIN2/3 (+) group. In the HPV+ group, 36 (72%) women had a benign histopathology result and 6 (12%) women had LSIL.

**Table 2:** Human papillomavirus test, cytology, and histopathology results of human papillomavirus+/cervical intraepithelial neoplasia2/3-group and human papillomavirus+/cervical intraepithelial neoplasia2/3+ group.

	HPV+/CIN2/3-(n=50)	HPV+/CIN2/3+(n=50)
HPV type		
HPV 16	24 (48)	33 (66)
HPV18	12 (24)	8 (16)
HPV56	4 (8)	2 (4)
HPV45	1 (2)	2 (4)
HPV31	1 (2)	0 (0)
HPV35	2 (4)	1 (2)
HPV42	1 (2)	0 (0)
HPV39	2 (4)	0 (0)
HPV66	1 (2)	1 (2)
HPV51	1 (2)	0 (0)
HPV11	0 (0)	1 (2)
HPV other type^*^	17 (34)	16 (32)
Multiple HPV	14 (28)	13 (26)
Cytology results		
None	12 (24)	7 (14)
Normal	14 (28)	9 (18)
ASCUS	16 (32)	9 (18)
LSIL	7 (14)	11 (22)
HSIL	1 (2)	10 (20)
ASC-H	0 (0)	4 (8)
Histopathological results		
None	8 (16)	0 (0)
Benign	36 (72)	0 (0)
LSIL	6 (12)	0 (0)
HSIL (CIN2/3)	0 (0)	50 (100)

ASCUS: atypical squamous cells of undetermined significance; ASC-H: atypical squamous cells, cannot exclude high-grade intraepithelial lesion; CIN: cervical intraepithelial lesion; LSIL: low-grade squamous intraepithelial lesion; HSIL: high-grade squamous intraepithelial lesion; HPV: human papillomavirus.

*HPV other type includes cases where the HPV type is not specified individually in the laboratory test results, but is only reported as “other type.” The HPV type results for women with multiple HPV type were presented separately in the table.

The study results showed that the oxidative stress was associated with HPV infection and the progression of HPV infection to the CIN2/3 (Table 3). The mean TAS level was higher in the control group (1.044±0.0959 mmol/L) than in both HPV+ group (0.931±0.1034 mmol/L) and CIN 2/3+ group (0.927±0.0928 mmol/L) (p<0.001). The median TOS level was lower in the control group than the CIN2/3+ group (4.570 [4.390–4.783] μmol/L vs. 4.780 [4.443–5.035] μmol/L, p<0.05). However, the IMA levels were similar in three groups (p=0.220).

**Table 3:** Total antioxidant status, total oxidant status, and ischemia-modified albumin levels of groups.

	HPV+ (n=50)	CIN2/3+ (n=50)	Control (n=50)	p-value
TAS, mmol Trolox Equiv./L	0.931±0.1034	0.927±0.0928	1.044±0.0959	<0.001^a^
TOS, μmol H_2_O_2_ Equiv./L	4.675 (4.450–4.928)	4.780 (4.443–5.035)	4.570 (4.390–4.783)	0.034^b^
IMA, ABSU	0.734 (0.588–0.825)	0.800 (0.619–0.846)	0.677 (0.568–0.818)	0.220

ABSU: absorbance unit; IMA: ischemia-modified albumin; TAS: total antioxidant status; TOS: total oxidant status; HPV: human papillomavirus.

^a^Comparison of groups 1 and 2 (p=0.854); comparison of groups 1 and 3 (p<0.001); comparison of groups 2 and 3 (p<0.001).

^b^Comparison of groups 1 and 2 (p=0.163); comparison of groups 1 and 3 (p=0.273); comparison of groups 2 and 3 (p=0.032).

## DISCUSSION

The study results showed the association between oxidative stress and cervical HPV infection and progression to high-grade cervical preinvasive lesions. The higher TOS level was observed in CIN2/3+ women than in the control group in the study population. The TAS levels were higher in the control group than in both HPV+ and CIN2/3+ groups. The IMA biomarker failed to predict the relationship between oxidative stress and HPV infection and cervical preinvasive lesions.

There are many studies reporting the association between oxidative stress and various malignancies such as colorectal, prostate, and ovarian cancer^14,15,16^. Previous reports also showed that the loss of equilibrium of reactive oxygen species and endogenous antioxidants contributes to the cervical cancer pathogenesis^4,5,17^. Zahra et al.^17^ reported the elevated levels of malondialdehyde and 8-hydroxy 2-deoxyguanosine, and the decreased level of glutathione, which is one of the leading antioxidant molecules in women with cervical cancer. In another study, the higher plasma disulfide levels and the lower plasma native thiol and total thiol levels were reported in cervical cancer cases than in healthy women^5^. In these studies, various oxidant and antioxidant biomarkers were measured individually with different detection methods to evaluate the effect of oxidative stress on carcinogenesis in cervical cells. Differently, we evaluated the oxidative stress status by measuring total oxidant and antioxidant status and found that the TOS and TAS levels were different in women with high-grade cervical preinvasive lesions and healthy women.

Although there are many studies in the literature reporting the association between cervical cancer and oxidative stress, there are few reports evaluating the impact of oxidative stress on HPV infection and the progression of this infection to premalignant lesions^6,7,8^. Lin et al.^6^ reported the association between the oxidative stress markers and HPV infection, persistence, and CIN. In their studies, the protein carbonyl levels were higher in women with HPV infection and persistence. They also reported elevated protein carbonyl levels in women with LSIL. Sezgin et al.^7^ found lower native thiol and total thiol levels, and higher disulfide levels in women with HSIL than in healthy controls. Similar to previous reports, we observed the association between oxidative stress and HPV infection and highgrade CIN. The TOS level was higher in CIN2/3+ women, and the TAS level was lower in both HPV+ and CIN2/3+ women than in the control. These findings suggest that interventions such as increasing dietary antioxidants may prevent the development of high-grade cervical preinvasive lesions and subsequent invasive cervical cancer due to HPV infection. There are many studies in the literature that support this idea. Studies conducted on HeLa cell culture, rats, and women with CIN have reported that dietary antioxidants such as resveratrol, curcumin, vitamin E, and vitamin D reduce the incidence of dysplasia, prevent carcinoma in situ, inhibit proliferation in cancer cells, induce apoptosis, and promote CIN regression^4^. Similarly, the studies showed that melatonin, another antioxidant, reduces recurrent genital infections and has antiproliferative, pro-apoptotic, anti-angiogenic, and antimetastatic properties in malignant cases^18,19^.

Nowadays, high-risk HPV testing has enabled the early diagnosis of cervical preinvasive lesions and cervical cancer. HPV testing could be used with and without cervical cytology in screening programs. However, discrepancies between the HPV test and cytology results could lead to clinical diagnostic difficulties^20^. New screening strategies are being developed to address this clinical problem. Lorenzi et al. reported that high-risk HPV testing combined with the biomarker p16/Ki-67 is a useful screening method for the detection of women at risk of cervical cancer^21^. The results of our study suggest that adding TAS/TOS level measurement to HPV testing could also improve the accuracy of screening tests.

Early diagnosis of cervical cancer is crucial not only for survival but also for future quality of life and fertility. As with other gynecological diseases^22^, early diagnosis can enable the administration of fertility-preserving treatment options. Evaluating the oxidative stress with TAS/TOS levels in HPV+ cases could help predict women at high risk of cervical cancer and enable early treatment.

Limited studies with conflicting results reported the predictive value of IMA, another oxidative stress biomarker, for cervical cancer and oxidative stress association^5,7^. Sezgin et al.^7^ demonstrated higher IMA levels in women with HSIL than in healthy women. However, in another study, the IMA levels of women with cervical cancer and the control group were not significantly different^5^. In our study, the IMA levels were similar in women with HPV infections, with CIN2/3, and healthy women. While the oxidative stress level associated with HPV infection and cervical preinvasive lesions may be sufficient to cause changes in TAS/TOS levels, it may not have caused severe hypoxia and acidosis enough to alter albumin’s metal-binding capacity. Therefore, no significant differences in IMA levels may have been demonstrated between the groups in the presented study.

This study is one of the few studies in the literature reporting the relationship between oxidative stress and cervical HPV infection and preinvasive lesions. The prospective study design and the assignment of CIN2/3 cases according to histopathological diagnosis are the other strengths of the research. However, the study has several limitations. The age difference between groups was a major limitation of the study. The mean age was significantly higher in both HPV+ and CIN2/3+ groups than in the control. Previous studies have shown that the oxidant and antioxidant biomarker levels change with age and menopausal status^23^. The effect of older age on oxidant and antioxidant levels may have influenced the study results. The higher rates of postmenopausal women in the HPV+ and CIN2/3+ groups compared to the control group may have influenced the study results, similar to age. The lack of knowledge about dietary habits was one of the limitations. The effect of dietary antioxidants on the analysis results could not be evaluated. Another limitation is that only one HPV test result of the participants was available. The study design did not include the longitudinal follow-up of the cases. Therefore, it is not known whether the HPV infection in the HPV+ group was a persistent infection or not.

## CONCLUSION

The serum TAS and TOS measurements revealed considerable oxidative damage in women with cervical HPV infection and high-grade preinvasive lesions compared with healthy women. These findings suggest that oxidative stress plays a role in the etiopathogenesis of HPV infection and progression to precancerous lesions of the cervix. Assessment of total oxidant and antioxidant status in clinical practice could guide the early identification and management of patients at high risk of cervical HPV infection and cancer progression. No significant association was demonstrated between IMA, another oxidative stress marker, and HPV infection and the presence of CIN2/3. This highlighted the importance of identifying appropriate oxidative stress biomarkers for the detection of cervical HPV infection, preinvasive lesions, and cancer prevention.

## Data Availability

The datasets generated and/or analyzed during the current study are available from the corresponding author upon reasonable request.
